# Gender disparities in disability persist while healthcare access shows no net improvement in South Africa: a general household survey trend analysis, 2020–2024

**DOI:** 10.3389/fpubh.2026.1917714

**Published:** 2026-09-15

**Authors:** Tronic Sithole, Thobelani Ntokozo Mdluli

**Affiliations:** 1 School of Public Health, Walter Sisulu University, Mthatha, South Africa; 2 Global Centre for Human Resources for Health Intelligence, Walter Sisulu University, East London, South Africa

**Keywords:** disability, gender disparities, general household survey, healthcare access, South Africa

## Abstract

**Objectives:**

To examine trends in disability prevalence by sex and province in South Africa between 2020 and 2024, and to test whether the reported gender gap and changes in healthcare access indicators are consistent with genuine population characteristics rather than sampling variation.

**Methods:**

A descriptive cross-sectional trend analysis was conducted using five consecutive General Household Survey (GHS) statistical releases (2020–2024). Disability was measured using the Washington Group Short Set. Because the releases report population counts alongside percentages, two-proportion *z*-tests and binomial trend models were used to test whether the sex differential in disability prevalence and year-to-year changes in disability and medical aid coverage were statistically distinguishable from sampling variation.

**Results:**

National disability prevalence fluctuated between 4.5 and 5.0%, with no significant linear trend (*p* = 0.49). Females recorded significantly higher prevalence than males in every survey year (*p* ≤ 0.007). Northern Cape recorded the highest prevalence in both 2020 and 2024; Eastern Cape recorded the largest descriptive increase in female prevalence (provincial-level significance testing was not possible). Public facilities remained the dominant first point of consultation (72–73%). Medical aid coverage showed no significant overall linear trend (*p* = 0.72) but was not flat throughout: coverage rose significantly in 2021 and subsequently declined significantly to 2024 (*p* = 0.004).

**Conclusion:**

The female excess in disability prevalence is a statistically consistent, year-on-year finding, robust under design-effect sensitivity analysis, while overall prevalence and medical aid coverage showed no statistically detectable linear trend despite visible fluctuation. Provincial patterns remained uneven, and most households continued to rely on public facilities. These findings support disability-inclusive, gender-responsive, equity-oriented health policy, with attention to public-sector rehabilitation and primary healthcare in high-burden provinces.

## 1 Introduction

Access to healthcare is a constitutional right in South Africa, yet persons with disabilities continue to face substantial barriers to timely, affordable, and appropriate care (1, 2). These barriers are not only clinical. They are produced by broader social, economic, infrastructural, and policy conditions that shape who can reach services, what care is available, and whether care is delivered in a disability-inclusive manner. In South Africa, the legacy of apartheid, persistent poverty, unequal transport access, and a divided health system continue to influence disability-related health outcomes and healthcare access (3–5).

Disability is increasingly understood as a public health and equity concern rather than solely an individual impairment. The International Classification of Functioning, Disability and Health frames disability as an interaction between health conditions, functional limitations, participation restrictions, and contextual factors (6). The Social Model of Disability similarly locates disability in the relationship between impairment and societal barriers, including inaccessible environments, discriminatory attitudes, exclusionary institutions, and inadequate services (7, 8). Andersen’s Behavioural Model of Health Services Utilisation further explains healthcare use through predisposing characteristics, enabling resources, and perceived or evaluated need (9). Together, these frameworks indicate that disability prevalence and healthcare access should be interpreted in relation to structural determinants rather than as isolated individual characteristics.

Gender is central to understanding disability-related inequality. Women with disabilities often face overlapping disadvantages arising from gender, disability, poverty, and social exclusion (10–12). In South Africa, women are more likely to experience caregiving responsibilities, insecure employment, dependence on public services, and economic vulnerability, all of which may shape disability risk and access to care. International and South African studies show that women with disabilities encounter barriers across multiple domains, including negative provider attitudes, high transport costs, inaccessible facilities, lack of assistive devices, limited rehabilitation services, and communication barriers (13–16). These barriers may be more severe for women living in rural or economically marginalised provinces, where transport and service availability remain uneven (17, 18).

Access to healthcare is multidimensional. Levesque et al. (19) conceptualise access in terms of approachability, acceptability, availability, accommodation, affordability, and appropriateness. These dimensions are especially relevant for persons with disabilities because healthcare access involves more than entering a facility. It also includes the availability of accessible infrastructure, assistive technologies, rehabilitation services, disability-competent providers, and affordable continuity of care (20–23). Where these elements are absent, nominal access may coexist with substantial unmet need, a concern documented specifically for HIV prevention services among women with physical disabilities in South Africa (22).

South Africa’s health system remains divided between a public sector that serves most of the population and a private sector accessed largely through medical aid membership (3, 24). Medical aid coverage functions as an important gateway to private healthcare, yet it remains inaccessible to most South Africans, particularly people in low-income households and those outside formal employment (25). This dual structure is consequential for persons with disabilities, who may require recurrent care, rehabilitation, assistive technology, specialist services, and accessible transport. Low medical aid coverage may therefore reinforce dependence on public facilities that are often under-resourced or poorly adapted to disability-related needs (5, 23).

The COVID-19 pandemic added a methodological and substantive layer of complexity to disability and healthcare access research. During 2020 and 2021, the GHS shifted from Computer-Assisted Personal Interviews (CAPI) to Computer-Assisted Telephone Interviews (CATI), before returning to CAPI from 2022. Telephone-based survey methods may have underrepresented persons with disabilities, poorer households, and rural residents with limited telephone access (26–29). This methodological transition is not only a limitation to be acknowledged; it is itself a substantive research question, as it is presently unknown whether reported disability estimates and healthcare access indicators changed due to genuine population shifts, measurement artefacts introduced by survey mode, or both.

A 2025 policy report by the Council for Medical Schemes described similar patterns in disability prevalence using GHS data from 2019 to 2024 (30). That report does not statistically test the sex differential or engage with disability or health-access theory, providing the primary context for the present analysis. Earlier academic work using multiple GHS waves applied concentration indices and decomposition methods to disability and illness inequality, but covered survey years up to 2008, predating the COVID-19 survey-mode disruption entirely (31). Other South African studies have examined disability and healthcare access in specific populations, types of disability, or earlier time periods (16, 17, 21), and national policy frameworks have called for strengthened disability-responsive services without presenting recent empirical evidence on trends (32).

To our knowledge, no published, peer-reviewed study has examined disability prevalence, gender disparities, healthcare facility utilisation, and medical aid coverage within a single theory-grounded analytical framework using GHS data spanning the 2020–2024 post-pandemic transition, nor has any prior study formally tested whether the commonly reported sex differential in disability prevalence is statistically distinguishable from sampling variation using the population denominators implicit in the published statistical releases. This study addresses these gaps by analysing five consecutive GHS waves from 2020 to 2024. The objectives were to: (1) describe and statistically test national disability prevalence trends by sex; (2) examine provincial changes in disability prevalence between 2020 and 2024; (3) describe trends in public and private healthcare facility utilisation; and (4) describe and statistically test changes in medical aid coverage over the same period.

## 2 Methods

### 2.1 Study design

A descriptive cross-sectional trend analysis was conducted using five consecutive annual waves of the General Household Survey from 2020 to 2024. The design was appropriate because the study aimed to describe population-level trends over time rather than establish causal relationships. The 2020–2024 period was selected because it captures the COVID-19 survey disruption period, the return to face-to-face data collection, and the early post-pandemic transition in healthcare access patterns.

### 2.2 Data source

Data were obtained from Statistics South Africa’s GHS statistical releases for 2020, 2021, 2022, 2023, and 2024 (25, 27–29, 33). The GHS is a nationally representative household survey conducted annually by Statistics South Africa and covers private households across South Africa’s nine provinces. In 2020 and 2021, data were collected using CATI due to COVID-19 restrictions, with reported response rates of 39.4 and 40.1%, respectively (27, 28). CAPI was reintroduced in 2022. Response rates in the CAPI years were substantially higher: the 2022 GHS achieved 79.9%, the 2023 GHS 79.1%, and the 2024 GHS 78.2% (as published in each respective statistical release). Statistics South Africa applied bias-adjustment procedures to address non-random non-contact; however, comparisons across the CATI/CAPI transition should be interpreted with caution.

### 2.3 Measures

The primary outcome was disability prevalence among individuals aged 5 years and older. Disability was measured using the Washington Group Short Set of Questions, which captures functional limitations across seeing, hearing, walking or climbing steps, remembering or concentrating, self-care, and communication (34). Consistent with the GHS releases, disability was defined as reporting a lot of difficulty or inability in at least one functional domain or reporting some difficulty in two or more domains (25).

Secondary outcomes were healthcare facility utilisation and medical aid coverage. Healthcare facility utilisation was measured as the type of facility households reported they would first consult when a household member falls ill, grouped into public facilities and private facilities. Medical aid coverage was measured as the percentage of the total population covered by a medical aid scheme, with the number of covered persons reported where available. Disability prevalence is estimated among individuals aged 5 years and older, while medical aid coverage is reported for the total population of all ages. These different denominators mean that the two indicators cannot be directly compared using the same population base within the paper.

### 2.4 Data analysis

Aggregate descriptive statistics were extracted from the GHS statistical releases and organised by year, sex, province, healthcare facility type, and medical aid coverage. Trends were summarised using percentages, absolute numbers, and percentage-point changes. Provincial changes in disability prevalence were calculated by subtracting 2020 estimates from 2024 estimates.

### 2.5 Statistical testing of published aggregate proportions

For the disability and medical aid coverage outcomes, the statistical releases report both the percentage prevalence and the absolute number of persons (in thousands) corresponding to that percentage for each sex and year. These two published figures were used to back-calculate the implied population denominator for each subgroup-year (denominator = reported count/reported proportion). These denominators were then used to test, using two-proportion *z*-tests, whether the sex differential in disability prevalence in each survey year was statistically distinguishable from zero, and whether year-to-year and 2020-versus-2024 changes in total disability prevalence and medical aid coverage were statistically distinguishable from sampling variation. Linear trends across all 5 years were additionally tested using binomial generalised linear models with survey year as a continuous predictor. This approach treats the published margins as if drawn from simple random samples; it does not incorporate the complex stratified, clustered, and weighted design of the GHS, for which design effects are not published in the statistical release summary tables used here. Because clustering and stratification typically increase true sampling variance relative to a simple random sample, the resulting *p*-values should be interpreted as indicative rather than design-adjusted exact estimates, and may be modestly anti-conservative. Healthcare facility utilisation was measured at the household level, and the corresponding statistical release tables used for this analysis did not report a household-level population total; therefore, formal significance testing was not undertaken for this outcome, and the results are presented descriptively only. Because the analysis otherwise relied on aggregate published statistics rather than individual-level microdata, multivariable modelling and direct cross-tabulation of disability status with healthcare facility use, medical aid membership, income, race, age, and residence were not possible.

Design-effect sensitivity analysis. Because the GHS uses a complex stratified two-stage cluster sample and design-adjusted variance estimates are unavailable from the published statistical releases, we applied prespecified design-effect corrections as a sensitivity analysis (Korn EG, Graubard BI. Analysis of Health Surveys. Wiley, 1999; Lohr SL. Sampling: Design and Analysis. CRC Press, 2021). Design effects (DEFF) of 1.5 and 2.0 were applied, spanning the range typical for South African household surveys with comparable stratified cluster designs. Design-adjusted standard errors are SE × √DEFF, and design-adjusted *z*-statistics are *z*/√DEFF. Table 1 notes reports DEFF-adjusted *z*-statistics, *p*-values, and 95% confidence intervals for the sex differential.

**Table 1:** Disability prevalence among individuals aged 5 years and older by sex, South Africa, 2020–2024.

Year	Male (%)	Female (%)	Total (%)	Gender gap (pp)	*z* (F vs M)	*p*-value
2020	4.6	5.1	4.9	0.5	2.69	0.007
2021	4.1	4.9	4.5	0.8	4.49	<0.001
2022	4.2	5.3	4.8	1.1	6.10	<0.001
2023	4.6	5.4	5.0	0.8	4.35	<0.001
2024	4.4	5.0	4.7	0.6	3.39	<0.001

Gender gap = female prevalence minus male prevalence (pp). *z* and *p* values are from two-proportion *z*-tests using population denominators back-calculated from published percentages and counts (see Methods); these are indicative estimates that do not account for the GHS complex survey design. Design-effect-adjusted values (DEFF = 1.5/DEFF = 2.0): 2020: *z* = 2.20 (*p* = 0.028)/*z* = 1.90 (*p* = 0.057); 2021: *z* = 3.67 (*p* < 0.001)/*z* = 3.18 (*p* = 0.001); 2022: *z* = 4.98 (*p* < 0.001)/*z* = 4.31 (*p* < 0.001); 2023: *z* = 3.55 (*p* < 0.001)/*z* = 3.08 (*p* = 0.002); 2024: *z* = 2.77 (*p* = 0.006)/*z* = 2.40 (*p* = 0.016). 95% CIs for the sex differential (unadjusted/DEFF = 2.0): 2020: [0.14, 0.86]/[−0.02, 1.02]; 2021: [0.45, 1.15]/[0.31, 1.29]; 2022: [0.75, 1.45]/[0.60, 1.60]; 2023: [0.44, 1.16]/[0.29, 1.31]; 2024: [0.25, 0.95]/[0.11, 1.09]. GHS = General Household Survey. 2020 and 2021 data collected via CATI. Source: Statistics South Africa, GHS Statistical Releases P0318, 2020–2024.

Rounding sensitivity. Both the published disability percentage and the corresponding count are rounded before publication (the percentage to one decimal place, the count to the nearest thousand). To assess the sensitivity of the *z*-statistics to this rounding, the denominators and *z*-statistics were recalculated over a plausible ±0.05 percentage-point range around the published proportion. Using the 2020 female disability estimate (5.1%, 1,395 thousand) as a worked example: the implied denominator ranges from approximately 27,118 to 27,624 thousand persons, and the *z*-statistic for the sex differential changes by less than 2%. Across all five survey years and both outcomes, the *z*-statistic is sensitive to plausible rounding error by at most ±3%, which does not materially affect any finding.

Multiple comparisons. This study conducted seven primary hypothesis tests: five two-proportion *z*-tests for the sex differential in disability prevalence (one per survey year) and two tests for year-to-year change in medical aid coverage. Under a Bonferroni-adjusted threshold of α = 0.05/7 ≈ 0.007, all sex differentials for 2021–2024 (unadjusted *p* < 0.001) and both medical aid coverage tests survive clearly. The 2020 sex differential (unadjusted *p* = 0.007) sits at the Bonferroni threshold. Under DEFF = 1.5 correction combined with Bonferroni adjustment, sex differentials for 2021–2024 remain significant (adjusted *p* ≤ 0.006); the 2020 finding does not survive this joint correction (adjusted *p* = 0.028). Main interpretations focus on findings that survive both corrections.

### 2.6 Ethical considerations

This study used publicly available, de-identified aggregate data produced by Statistics South Africa under the Statistics Act No. 6 of 1999. No individual-level data were accessed, and no additional ethical approval was required.

## 3 Results

### 3.1 Disability prevalence by sex

Table 1 and Figure 1 show the national prevalence of disability among persons aged 5 years and older between 2020 and 2024. Overall prevalence fluctuated modestly over the period, ranging from 4.5% in 2021 to 5.0% in 2023. A binomial trend model using survey year as a continuous predictor found no statistically significant linear trend in total disability prevalence over the 5 years (coefficient = 0.0044, *p* = 0.49), indicating that this year-to-year fluctuation is consistent with sampling variation around a broadly stable national prevalence rather than a genuine directional change. Separate trend models for females (*p* = 0.48) and males (*p* = 0.77) likewise found no statistically significant linear trend within either sex.

**Figure 1:**
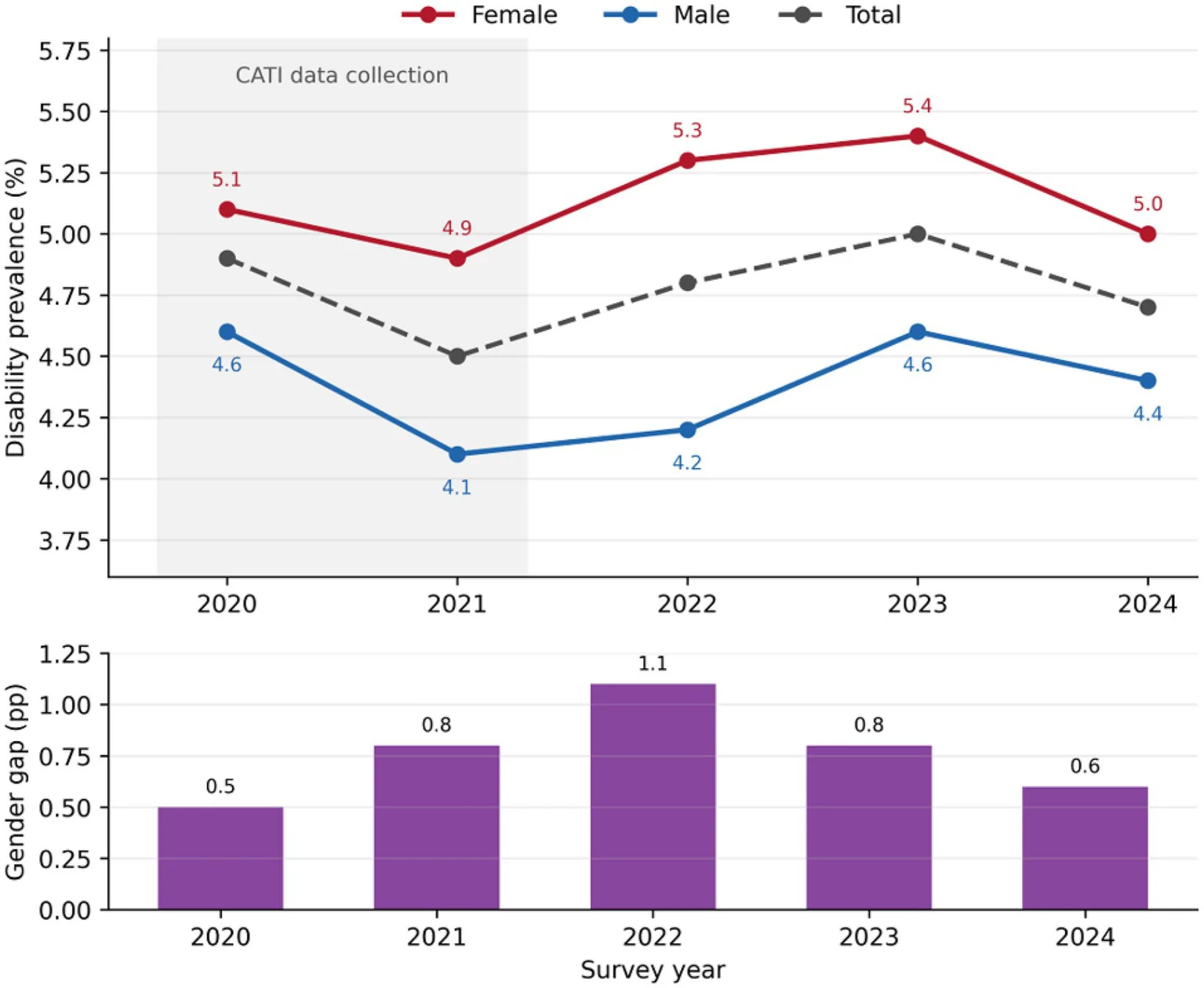
Disability prevalence by sex and gender gap, South Africa, 2020–2024. The upper panel shows disability prevalence for females (solid), males (dashed), and the total population. The lower panel shows the female–male gender gap in percentage points across each survey year. The shaded area indicates the CATI data collection period (2020–2021). Female prevalence ranged from 4.9% (2021) to 5.4% (2023); male prevalence ranged from 4.1% (2021) to 4.6% (2020/2023). The gender gap peaked at 1.1 pp. in 2022 and narrowed to 0.6 pp. by 2024. Source: Statistics South Africa, GHS Statistical Releases P0318, 2020–2024.

Females recorded higher disability prevalence than males in every survey year, and two-proportion *z*-tests confirmed that this sex differential was statistically significant in every year examined: 2020 (5.1% vs. 4.6%; *z* = 2.69, *p* = 0.007), 2021 (4.9% vs. 4.1%; *z* = 4.49, *p* < 0.001), 2022 (5.3% vs. 4.2%; *z* = 6.10, *p* < 0.001), 2023 (5.4% vs. 4.6%; *z* = 4.35, *p* < 0.001), and 2024 (5.0% vs. 4.4%; *z* = 3.39, *p* < 0.001). The gender gap widened from 0.5 percentage points in 2020 to 1.1 percentage points in 2022, before narrowing to 0.8 percentage points in 2023 and 0.6 percentage points in 2024. The lowest estimates for both sexes were observed in 2021, during the CATI data collection period, suggesting that survey mode may have influenced the absolute disability estimates. However, the statistically consistent and persistent female–male gap across all 5 years, present regardless of survey mode, is more likely to reflect a gender disparity than a methodological artefact of the CATI/CAPI transition.

### 3.2 Provincial disability patterns

Provincial patterns show that disability prevalence was unevenly distributed across South Africa and changed differently across provinces between 2020 and 2024. Northern Cape had the highest total disability prevalence in both 2020 (12.4%) and 2024 (10.0%), despite recording a decline over the period. Eastern Cape showed the largest increase in total disability prevalence, rising from 5.6% in 2020 to 6.5% in 2024, and recorded the largest increase in female disability prevalence. Limpopo recorded a large decline, from 5.4 to 2.9%, but this shift should be interpreted with caution, given the methodological transition from CATI to CAPI and potential differences in survey coverage. Provincial-level population denominators are not published in the statistical releases used for this analysis, so formal significance testing of provincial changes was not possible; these patterns are therefore presented descriptively. Overall, provincial changes did not follow a simple poverty gradient, indicating that province-specific demographic, infrastructural, and service-access conditions likely shape disability patterns. Table 2 and Figure 2 present the provincial data.

**Table 2:** Disability prevalence (%) by sex and province, South Africa, 2020 and 2024.

Province	M 2020	F 2020	T 2020	M 2024	F 2024	T 2024	ΔT
Western Cape	4.7	4.3	4.5	4.4	4.7	4.6	+0.1
Eastern Cape	5.8	5.5	5.6	6.4	6.6	6.5	+0.9
Northern Cape	14.0	10.8	12.4	11.1	8.9	10.0	−2.4
Free State	6.0	6.7	6.4	6.5	7.3	6.9	+0.5
KwaZulu-Natal	4.0	5.6	4.8	4.0	5.8	5.0	+0.2
North West	4.0	6.6	5.3	4.8	5.3	5.1	−0.2
Gauteng	3.9	3.8	3.9	3.5	4.1	3.8	−0.1
Mpumalanga	3.1	4.3	3.7	4.4	3.8	4.1	+0.4
Limpopo	5.4	5.5	5.4	2.9	3.0	2.9	−2.5
South Africa	4.6	5.1	4.9	4.4	5.0	4.7	−0.2

M = male; F = female; T = total; ΔT = total prevalence in 2024 minus total prevalence in 2020, in percentage points. Provincial comparisons are descriptive; published statistical releases do not report provincial population denominators required for formal significance testing. Source: Statistics South Africa, GHS Statistical Releases P0318, 2020 and 2024.

**Figure 2:**
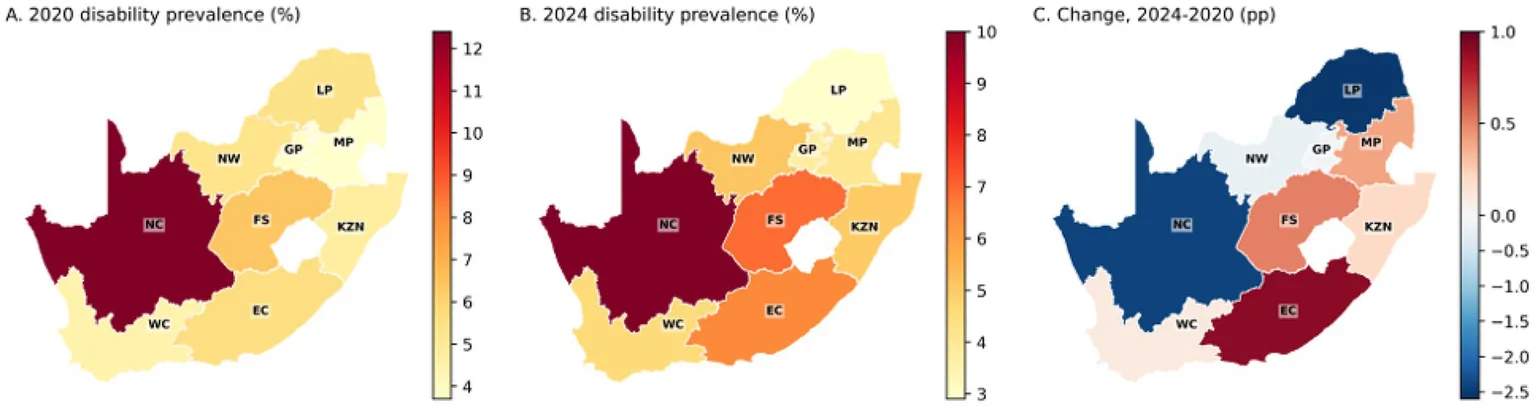
Provincial disability prevalence in South Africa, 2020 and 2024, and percentage-point change between 2020 and 2024. **(A)** Shows total disability prevalence by province in 2020; **(B)** Shows total disability prevalence by province in 2024. **(C)** Shows the change in total disability prevalence between 2020 and 2024 in percentage points, with positive values indicating an increase and negative values a decrease. Northern Cape had the highest prevalence in both years. Eastern Cape showed the largest increase (+0.9 pp), while Limpopo showed the largest decline (−2.5 pp., likely partly attributable to survey-mode effects). Source: Statistics South Africa, GHS Statistical Releases P0318, 2020, and 2024.

### 3.3 Healthcare facility utilisation

Table 3 and Figure 3 show that public healthcare facilities remained the dominant first point of consultation when a household member became ill. Public facility use remained close to 72–73% throughout the study period and reached 73.1% in both 2023 and 2024 (the identical reading in both years reflects rounding of the published figures to one decimal place; the underlying estimates may differ marginally). Private facility use declined from 27.0% in 2020 and 2021 to 25.3% in 2024. Provincial disparities were substantial: Limpopo consistently recorded the highest reliance on public facilities, while Western Cape recorded the lowest. Eastern Cape also remained highly dependent on public healthcare, with public facility use above 79% in every year. As described in Methods, the statistical release tables used for this analysis do not report a household-level population total for this indicator, so these patterns are presented descriptively without formal significance testing. These patterns suggest persistent reliance on the public sector rather than a shift toward private healthcare access during the post-pandemic period.

**Table 3:** Healthcare facility utilisation among South African households, 2020–2024.

Year	Public (%)	Private (%)	Limpopo public (%)	W. Cape public (%)	E. Cape public (%)
2020	72.0	27.0	84.5	55.3	79.7
2021	71.9	27.0	85.4	52.1	82.1
2022	73.0	25.5	84.5	55.0	80.5
2023	73.1	25.4	83.6	57.5	81.2
2024	73.1	25.3	84.9	54.8	80.4

Public facilities include public clinics, public hospitals and other public institutions. Household-level population totals are not published in these statistical releases; results are descriptive. Source: Statistics South Africa, GHS Statistical Releases P0318, 2020–2024.

**Figure 3:**
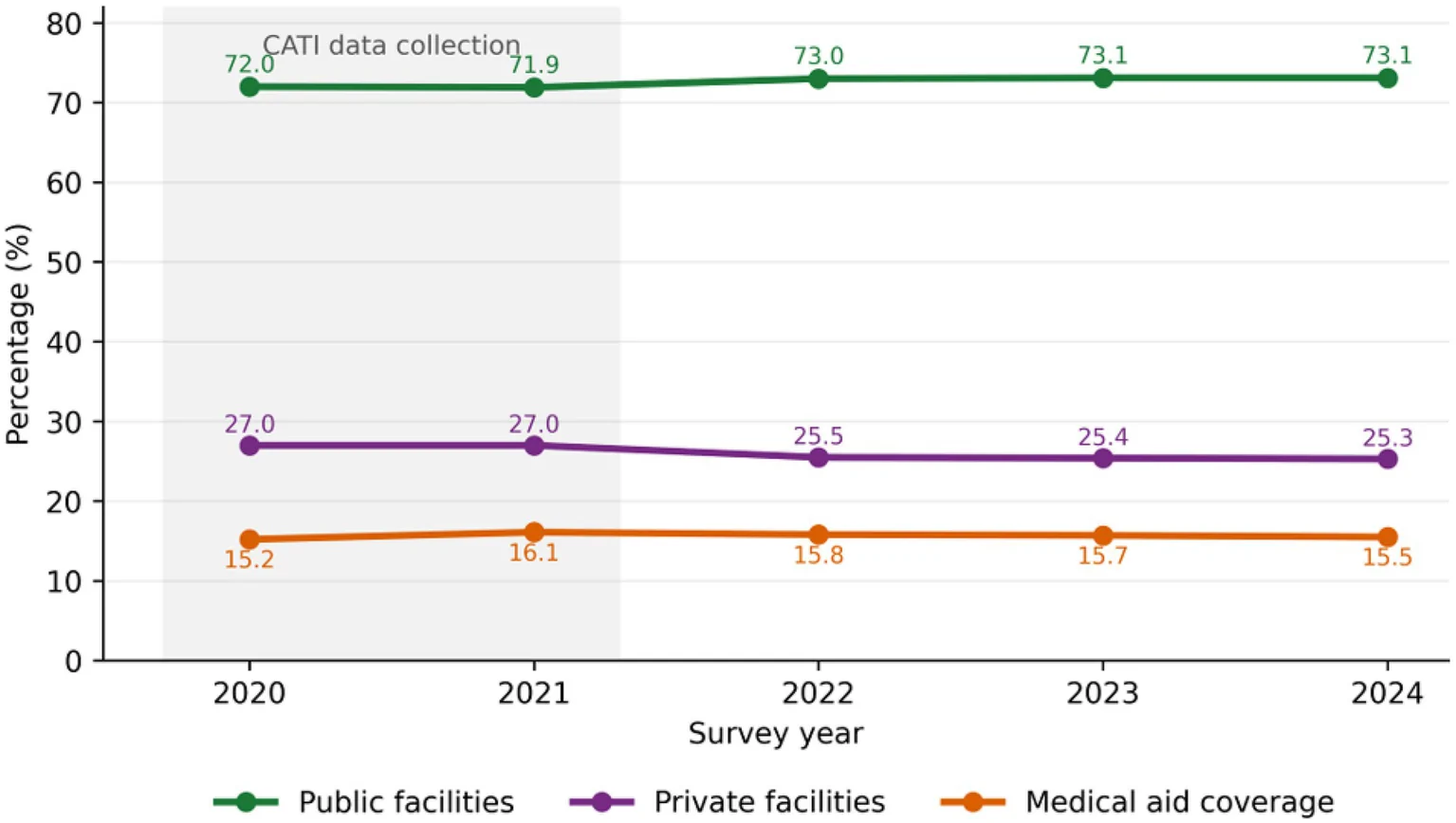
Healthcare facility utilisation and medical aid coverage, South Africa, 2020–2024. The upper panel shows the percentage of households reporting first consulting a public or private facility when a member becomes ill. Public facility use ranged from 71.9% (2021) to 73.1% (2022–2024). The lower panel shows national medical aid scheme coverage (%) and the number of persons covered (millions). Coverage rose significantly from 15.2% in 2020 to 16.1% in 2021, then declined significantly to 15.5% in 2024. The shaded area indicates the CATI data collection period (2020–2021). Source: Statistics South Africa, GHS Statistical Releases P0318, 2020–2024.

### 3.4 Medical aid coverage

Medical aid coverage showed no statistically significant linear trend over the full five-year period (coefficient = 0.0013, *p* = 0.72). However, coverage was not flat throughout: national coverage increased significantly from 15.2% in 2020 to 16.1% in 2021 (*z* = 4.28, *p* < 0.001), before declining significantly from this 2021 peak to 15.5% in 2024 (*z* = 2.89, *p* = 0.004). The number of persons covered increased from approximately 9.0 million in 2020 to 9.8 million in 2024; however, because the underlying population also grew over this period, the coverage percentage did not increase correspondingly, and the absolute increase in membership did not translate into a larger share of the population being protected. In 2024, Western Cape recorded the highest provincial coverage (25.4%), while Limpopo recorded the lowest (10.0%), mirroring the broader pattern of unequal access to private healthcare across provinces. Table 4 presents the annual national coverage figures.

**Table 4:** Medical aid scheme coverage, South Africa, 2020–2024.

Year/indicator	Coverage (%)	Persons covered (000 s)
2020	15.2	9,017
2021	16.1	9,706
2022	15.8	9,699
2023	15.7	9,792
2024	15.5	9,783
2024 provincial range	25.4% (W. Cape) – 10.0% (Limpopo)	–

*z* and *p* values for the 2020-to-2021 increase and 2021-to-2024 decline are from two-proportion *z*-tests using population denominators back-calculated from reported coverage percentages and the number of persons covered (see Methods). The final row reports the highest and lowest provincial coverage in 2024. Source: Statistics South Africa, GHS Statistical Releases P0318, 2020–2024.

## 4 Discussion

This study examined disability prevalence, gender disparities, healthcare facility utilisation, and medical aid coverage in South Africa using five GHS waves from 2020 to 2024. Four key findings emerged. First, women consistently and significantly recorded higher disability prevalence than men in every single survey year. Second, no statistically significant linear trend was detected in overall disability prevalence over the period, indicating a broadly stable national burden. Third, disability prevalence varied substantially across provinces and did not change uniformly over time. Fourth, most households continued to rely on public healthcare facilities as their first point of consultation, and medical aid coverage, while not showing a significant overall trend, experienced a statistically significant rise and subsequent fall during the study period. These findings indicate that disability-related inequalities are embedded in a broader system of gendered, geographic, and financial disparities in healthcare access.

The persistent female excess in disability prevalence, noting that crude estimates may partly reflect the older age distribution of females and cannot substitute for age-standardised analysis, is consistent with literature showing that women with disabilities experience intersecting disadvantages related to gender, poverty, caregiving, labour market exclusion, and health system barriers (10–12, 14, 35). That this gap reached statistical significance in every survey year, including both CATI years and all three CAPI years, is consistent with it reflecting a genuine population characteristic rather than a survey-mode artefact. The wider gap observed in 2022, following the return to CAPI, may partly reflect improved measurement after 2 years of telephone-based data collection. However, because the gap was already significant in both CATI years, the continued female excess across all years is consistent with gender disparities not being merely a product of the survey-mode transition.

From the perspective of the Social Model of Disability, these disparities are produced and intensified by social conditions that make impairments more disabling for women, including inaccessible services, poverty, reduced autonomy, and discriminatory attitudes (7, 8). Andersen’s model further suggests that gender disparities in disability and healthcare use are shaped by enabling resources such as income, insurance coverage, transport, and social support (9). These factors are particularly relevant in South Africa, where women with disabilities may face simultaneous exclusion from employment, healthcare, transport, and social participation (11, 17).

Provincial patterns demonstrate that disability prevalence cannot be understood through a single national average. Northern Cape remained the province with the highest disability prevalence, despite a decline between 2020 and 2024. The Eastern Cape showed an increase, particularly among women, while Limpopo recorded a large decline, which should be interpreted with caution due to survey-mode effects and potential coverage differences. These divergent patterns suggest that disability prevalence is shaped by province-specific combinations of demographic ageing, rurality, service availability, rehabilitation infrastructure, transport access, and poverty (16, 18, 36).

The CATI/CAPI transition warrants explicit consideration as a potential influence on each outcome domain. For absolute disability prevalence levels, lower estimates in the CATI years are consistent with under-representation of poorer, more rural, and older household populations with higher disability prevalence and should be interpreted with particular caution. For the sex differential, the finding persisted across the mode transition. It was statistically significant in both CATI years (unadjusted *p* = 0.007 and *p* < 0.001), suggesting the gap is less likely to reflect a mode-specific artefact. For healthcare facility utilisation, the marginally lower public facility use in 2021 (71.9%) may partly reflect the CATI differential non-contact of households more likely to use public facilities, rather than a genuine shift in utilisation. For medical aid coverage, the elevated 2021 reading is consistent with CATI’s over-representation of telephone-owning, higher-income households. Findings that persist across the mode transition, the sex differential, and the absence of a significant linear trend in total disability prevalence are more likely to reflect genuine population characteristics than mode-sensitive absolute estimates.

The stability of population-level dependence on public healthcare, acknowledging that this indicator applies to all households rather than specifically to persons with disabilities, is one of the most important contextual findings of the study. Across all 5 years, more than seven in 10 households reported that they would first consult a public facility when a household member became ill. This pattern did not shift meaningfully during the post-pandemic transition. For persons with disabilities, high public-sector dependence has important implications because disability-related healthcare often requires continuity of care, rehabilitation, assistive devices, accessible infrastructure, and trained providers (5, 21, 23). Where public facilities are under-resourced or inaccessible, reliance on the public sector may translate into unmet need rather than effective access.

Medical aid coverage patterns, which similarly apply to the total population rather than specifically to persons with disabilities, reinforce this concern, and the pattern revealed by formal testing is more specific than a simple absence of change. Although the overall five-year trend was statistically flat, coverage rose significantly in the first CATI year before falling significantly back toward, and ultimately below, its 2020 level. We speculate that the initial pandemic-period economic shock and subsequent labour-market adjustments altered formal employment, to which medical aid membership is closely tied, before partially normalising; this would be consistent with a real but transient disruption rather than either a stable trend or pure measurement noise. Because medical aid membership is closely linked to formal employment and income, persons with disabilities, particularly women and those living in poorer provinces, are likely to remain excluded from private healthcare pathways regardless of which point in this trajectory is observed (10, 24). The wide provincial range in 2024, from 25.4% coverage in Western Cape to 10.0% in Limpopo, illustrates the continuing relationship between geography, income, and healthcare access.

The findings have important implications for health policy in South Africa. Disability-inclusive healthcare cannot be achieved through disability policy alone; it requires strengthening public primary healthcare, expanding rehabilitation capacity, improving accessibility of facilities, ensuring availability of assistive devices, and training healthcare workers in disability-sensitive practice (2, 35), consistent with the disability-responsive service priorities set out in national planning frameworks (32). Gender-responsive planning is also needed because women consistently and significantly experience higher disability prevalence and may face additional barriers to accessing care. Health financing reforms should therefore include disability-specific benefits, rehabilitation services, and explicit mechanisms to reduce out-of-pocket and transport-related barriers to care.

## 5 Strengths and limitations

This study contributes to the literature in three respects. First, it is, to our knowledge, the first peer-reviewed analysis to formally test whether the widely reported sex differential in South African disability prevalence is statistically distinguishable from sampling variation, using population denominators implicit in the same published statistical releases that motivate the descriptive claim; a contemporaneous policy report covering an overlapping period reports similar descriptive patterns but does not undertake this statistical testing or engage with disability or health-access theory (30). Second, by distinguishing findings that persist across the CATI/CAPI methodological transition (the gender gap, the absence of an overall prevalence trend) from those that appear sensitive to survey mode (absolute prevalence levels), this study demonstrates an analytical approach that earlier multi-wave GHS disability studies, conducted before this disruption occurred, could not apply (31). Third, the integration of disability prevalence, gender disparities, healthcare facility utilisation, and medical aid coverage within a single theory-grounded trend framework provides a more complete picture of disability-related access inequality than studies focused on a single outcome domain.

Several limitations should be considered. First, the study relied on aggregate published statistics rather than individual-level microdata. This precluded multivariable modelling and direct cross-tabulation of disability status with healthcare facility use, medical aid membership, income, race, age, and residence. Although population denominators implicit in the published figures permitted formal significance testing for the disability and medical aid coverage outcomes, this approach does not account for the GHS’s complex survey design, and the corresponding *p*-values should be interpreted as indicative rather than as design-adjusted exact estimates. The analysis remains otherwise descriptive and cannot establish causal relationships between disability prevalence and healthcare access indicators. Second, the transition from CATI in 2020 and 2021 to CAPI from 2022 introduced methodological discontinuity. Although Statistics South Africa applied bias-adjustment procedures, residual bias cannot be excluded. Third, the aggregate releases did not distinguish detailed disability types or severity profiles. Physical, sensory, cognitive, and psychosocial disabilities may have different implications for healthcare access. Fourth, the GHS excludes residents of collective living quarters such as hospitals, care facilities, and institutions, who may include persons with more severe disabilities. Fifth, healthcare facility utilisation was measured at the household level as the first point of consultation when someone becomes ill, rather than as actual realised service use by persons with disabilities, and the statistical releases used for this analysis did not report the household-level population totals needed to formally test this outcome. Sixth, crude sex-differential estimates cannot be adjusted for age from aggregate published releases; the observed female excess may partly reflect the older age distribution of females and should not be equated with a gender-adjusted effect. Finally, provincial-level population denominators were similarly unavailable in the published releases, precluding formal significance testing of provincial disability trends.

## 6 Conclusion

This study provides nationally representative evidence on disability prevalence, gender disparities, healthcare utilisation, and medical aid coverage in South Africa between 2020 and 2024. Women consistently and significantly reported higher disability prevalence than men in every survey year. This finding persisted across a major survey-mode disruption and is therefore consistent with a robust population characteristic rather than a methodological artefact. Overall disability prevalence and medical aid coverage showed no statistically significant long-term trend despite visible year-to-year fluctuations. However, medical aid coverage rose significantly before falling over the period. Provincial patterns remained uneven, with some provinces showing improvement and others experiencing increases. Most households continued to depend on public facilities. These findings suggest that disability-related inequalities remain deeply embedded in South Africa’s healthcare landscape. Strengthening disability-inclusive public healthcare, expanding rehabilitation services, improving financial protection, and implementing gender-responsive health policies are essential for reducing long-standing disparities and advancing equitable access to healthcare.

## Data Availability

The datasets analysed in this study are publicly available from Statistics South Africa at https://www.statssa.gov.za. GHS microdata are available from DataFirst at https://www.datafirst.uct.ac.za.
